# Omega-6 oxylipins generated by soluble epoxide hydrolase are associated with knee osteoarthritis[S]

**DOI:** 10.1194/jlr.P085118

**Published:** 2018-07-09

**Authors:** Ana M. Valdes, Srinivasarao Ravipati, Petros Pousinis, Cristina Menni, Massimo Mangino, Abhishek Abhishek, Victoria Chapman, David A. Barrett, Michael Doherty

**Affiliations:** Academic Rheumatology,* Nottingham City Hospital, Nottingham, United Kingdom; Department of Twin Research,§ King’s College London, St Thomas’ Hospital, London, United Kingdom; National Institute for Health Research Nottingham Biomedical Research Centre,† University of Nottingham, Nottingham, United Kingdom; Centre for Analytical Bioscience, School of Pharmacy,** University of Nottingham, Nottingham, United Kingdom; School of Life Sciences,†† University of Nottingham, Nottingham, United Kingdom; Arthritis Research UK Centre of Excellence for Pain,§§ University of Nottingham, Nottingham, United Kingdom

**Keywords:** lipids, arachidonic acid, eicosanoids, inflammation, methods/high-performance liquid chromatography, lipidomics

## Abstract

Omega-6 FAs are inflammatory mediators that are increased in joints with osteoarthritis (OA), but their association with OA progression is not yet well defined. To investigate the relationship between omega-6 FAs and knee OA, we measured with LC-MS the levels of 22 omega-6 lipids (arachidonic acid, linoleic acid, and 20 oxylipins) in synovial fluid (SF) from 112 knees of 102 individuals (58 with knee OA; 44 controls). We hypothesized that oxylipin metabolites would increase in OA knee SF and with radiographically progressive disease. We validated results by comparing samples from affected and unaffected knees in 10 individuals with unilateral OA. In adjusted analysis, SF levels of three omega-6 oxylipins [prostaglandin D2, 11,12-dihydroxyeicosatrienoic acid (DHET), and 14,15-DHET] were associated with OA. Of these, 11,12-DHET and 14,15-DHET were higher in affected versus unaffected knees of people with unilateral disease (*P* < 0.014 and *P* < 0.003, respectively). Levels of these and 8,9-DHET were also associated with radiographic progression over 3.3 years in 87 individuals. Circulating levels of all three were associated with gene variants at the soluble epoxide hydrolase enzyme. Lipidomic profiling in SF identified an additional inflammatory pathway associated with knee OA and radiographic progression.

Osteoarthritis (OA) is the most common form of arthritis, and inflammation of the synovium (synovitis) is often evident in affected joints (1). Synovitis has been implicated as a cause of cartilage loss (2, 3) indicating that the inflammatory processes that take place in the OA synovium may be important contributors to disease progression (3). It has been assumed that the main drivers for this process are pro-inflammatory cytokines and prostaglandins (PGs) [e.g., see (4)], the latter being omega-6 pro-inflammatory mediators.

In addition to the role of PGs, several studies have implicated omega-6 PUFAs, which can be pro- or anti-inflammatory, in the pathogenesis of OA. A low ratio of omega-6/omega-3 PUFAs reduces the expression of the cartilage degeneration enzyme matrix metalloproteinase 13 and reduces adjuvant-induced arthritis in rats (5). In a murine model of OA, serum levels of omega-6 PUFAs [arachidonic acid (AA), eicosadienoic acid, γ linoleic acid (LNA), and dihomo-γ-linolenic acid] correlated positively with OA, impaired healing, and inflammatory adipokines (6). In humans, a positive association between the plasma omega-6 PUFAs, AA, and synovitis has been reported (7) and increased levels of AA have been found in the infrapatellar fat in knee OA compared with controls (8). PUFAs derived either from omega-6 (LNA, dihomo-γ-linolenic acid, AA) or omega-3 PUFAs are substrates for a number of different enzymes that generate biologically active oxygenated metabolites known as oxylipins (9).

Oxylipins are generated via the cyclooxygenase (COX), lipoxygenase (LOX), and cytochrome P450 pathways (10, 11). The COX pathway produces PGs and thromboxanes. The LOX pathway, which includes 5-LOX, 12-LOX, and 15-LOX, produces leukotrienes and HETEs from AA, as well as HODEs from LNA. The cytochrome 450 pathway predominantly produces epoxyeicosatrienoic acids (EETs), dihydroxyeicosatrienoic acids (DHETs), and HETEs from AA, and epoxyoctadecamonoenoic acids and dihydroxyoctadecenoic acid from LA (9, 11). The role of the levels of these additional synovial fluid (SF) omega-6 FAs in OA and OA progression has not been examined before.

We hypothesized that levels of omega-6 hydroxy or epoxy metabolites (oxylipins) will be increased in SF from OA knees compared with nonOA control knees in healthy volunteers and in OA knees compared with unaffected knees of individuals with unilateral knee OA. We further hypothesized that levels of these metabolites may increase in knees that progress to more severe radiographic OA. To test this, we quantified levels of 22 n-6 PUFAs, including LNA AA, and 20 n-6 PUFA oxylipins, in SF. The objectives of the study were, first, to assess whether levels of omega-6 lipids are different between OA and controls and then, to investigate whether these lipids are involved in radiographic progression.

## MATERIALS AND METHODS

### Osteoarthritis case-control cohort

A total of 102 individuals (44 nonOA controls and 58 with knee OA) were recruited from existing databases of previous OA studies at the University of Nottingham. Approval for recruitment was obtained from the research ethics committees of Nottingham City Hospital and North Nottinghamshire. All participants provided written informed consent. Bilateral knee radiographs (a single anteroposterior semi-flexed weight-bearing view using a Rosen template to control knee flexion and foot external rotation and 30° flexion skyline patellofemoral views) of index knee or hip OA cases were obtained at two time points and scored for features of OA by a single observer using the Kellgren and Lawrence (K/L) radiographic grade for the tibiofemoral and patella femoral compartments of each knee (12). Individuals also donated a blood sample (from which plasma was extracted) and an SF sample of 0.5 ml or more from one or both knees. These were stored at −80°C for further analysis. All individuals with OA had both radiographic signs of OA (K/L grade ≥2) and pain lasting 15 or more days in the past month as per American College of Rheumatology diagnostic guidelines (13).

SF samples were collected from 102 individuals (44 healthy volunteers and 58 affected knee OA participants) as previously described (14). Briefly, SF samples were microcentrifuged and underwent rapid freezing after the sample was taken. Samples were not treated before storage. The SF sample was then analyzed without further treatment (single aliquot).

### Plasma samples

Individuals from the same study also donated a blood sample from which plasma was extracted and stored for further analysis. Lipidomic analyses were carried out to determine plasma concentration levels of AA, linoleic acid, and the four hydroxyeicosatetraenoic acids.

In addition, circulating levels of total omega-3 and omega-6 in the plasma samples were measured using NMR metabolomic profiling by Nightingale Health, Finland (https://nightingalehealth.com), from fasting plasma samples using 500 MHz and 600 MhH proton NMR spectroscopy as previously described (15).

### Osteoarthritis progression

Participants in the baseline assessment were invited mean 3.25 years later (on average) to have a second radiographic and clinical assessment. Eighty-seven participants agreed to take part and had bilateral knee X-rays (identical protocol to that used at baseline) graded by the same observer as at baseline, blind of baseline film status. For the present study, we defined radiographic progression as a change of one or more in K/L grade for tibiofemoral OA. Individuals with a K/L grade of 4 at baseline were excluded from this analysis. Individuals without OA changes at baseline but with a higher radiographic score at follow-up (e.g., from K/L grade 1 to 2, and from K/L grade 0 to 1) were included and defined to have progression of OA.

### Knee effusion

The accumulation of excess SF in or around the knee joint is common among people with knee OA (16). In knee OA patients, clinical signs of effusion are significantly associated with inflammatory serum biomarkers (17). Therefore, we used the clinical assessment effusion performed at baseline as an indicator of inflammation of the joint to adjust for our analyses on structural progression.

### NSAID use

Individuals were asked about current prescription and nonprescription medication and were classified as taking nonsteroidal anti-inflammatory drugs (NSAIDs) if they reported taking any of the following: arthrotec, celebrex, diclofenac, ibuprofen, meloxicam, naproxen, or piroxicam. None of the participants reported taking other prescription NSAIDs such as indomethacin, nabumetone, etodolac, and tenoxicam.

### Anti-oxidant vitamins

Because some of the omega-6 compounds might generate from nonenzymatic pathways, we also extracted information from the questionnaires on use of multivitamins and anti-oxidant vitamins, and in secondary analyses, adjusted for use of these vitamins, including this binary variable as a covariate in the regression models.

### Oxylipin analysis method

The LC-MS/MS method used for eicosanoid analysis in human serum samples, based on the method previously developed (18) (11), was performed using fully extracted calibration standards for each of the analytes. Measured concentrations of the 22 omega-6 lipids listed in Table 1 were detectable in each sample and are corrected for sample volume where appropriate.

#### Equipment.

The HPLC system used was a Shimadzu series 10AD VP LC system (Shimadzu, Columbia, MD). The HPLC column used was ACE C18 (150 × 2.1 mm ID 3 µm particle size) with a guard column (Security Guard Cartridges ACE 3 C18 for ID 150 × 2.1 mm column). Mobile phase A was 0.02% formic acid in methanol:acetonitrile (1:4, v/v); mobile phase B was 0.02% formic acid in 100% water. The starting flow rate was 200 µl/min. Strata-X polymeric SPE column (200 mg/6 ml) was purchased from Phenomenex, Macclesfield, UK. The evaporator used was a Jouan centrifugal evaporator (Saint-Herblain, France). The MS system used was an Applied Biosystem MDS SCIEX 4000 Q-Trap hybrid triple-quadrupole-linear ion trap mass spectrometer (Applied Biosystem, Foster City, CA) equipped with an ESI interface.

Standards for all compounds were purchased from Cayman Chemicals (Ann Arbor, MI).

One batch of blank human plasma (for the OA case-control) or serum (for the TwinsUK study) acted as an analytical quality control used to confirm the day-to-day accuracy/precision of the method during the analysis of each batch of sample analysis.

#### Extraction protocol for samples.

Samples were stored at −80°C before analysis. Internal standards (100 µl of 2-AG-d8 (10 µM) and 15 µl of AEA-d8 (28 µM), 10 µl of PGF2a-EA-d4 (2.49 µM), 10 µl of AA-d8 (1 µM), 10 µl of PGD2-d4 (1 µM), and 10 µl of 15-HETE-d8 (7.6 µM) were added to each sample or blank sample (0.4 ml water) along with 2 µl of formic acid (98% v/v) and 5 µl of 0.5% w/v of an antioxidant butylhydroxytoluene (BHT) solution in ethanol. Samples were homogenized in micro centrifuge tubes with the addition of 900 µl ethanol, followed by a slow vortex stage (10 min), and centrifuged (13,000 *g*, 10 min, 4°C). The 100 µl supernatants were transferred to micro centrifuge tubes and diluted with an equal amount of 100% isopropanol prior to chemical analysis. Pooled quality controlled samples were prepared by combining 20 µl from each extract of individual SF samples.

Samples were homogenized in micro centrifuge tubes with the addition of 900 µl ethanol, followed by a slow vortex stage (10 min) and centrifuged (13000 *g*, 10 min, 4°C). The supernatants were transferred to glass tubes and diluted by the addition of 3 ml water. The diluted supernatants were loaded to the Strata-X polymeric SPE column (200 mg/6 ml) that had been preconditioned with 100% ethanol (2 ml) and 25% ethanol (4 ml). The SPE cartridge was then washed with distilled water (10 ml) and 25% ethanol (5 ml) and was allowed to run it dry. Then the eicosanoids were eluted from the column with ethyl acetate containing 0.0002% BHT (5 ml) and were dried in a centrifugal evaporator. The samples were reconstituted in 50% ethanol (100 µl) and transferred to an auto sampler vial prior to LC-MS/MS analysis. The injection volume was 20 µl. Quantification was performed using fully extracted calibration standards for each of the analytes. Quantification was performed using Analyst 1.4.1 (SCIEX LP, Ontario Canada; sciex.com/products/software/analyst-software). Identification of each compound in serum samples was confirmed by LC retention times of each standard and precursor and product ion *m/z* ratios. The peak area of each analyte was compared with a known amount of standard to determine the amount of target compound present.

### Statistical analysis

Concentrations of all omega-6 lipids were log-transformed for analysis in order to achieve a normal distribution necessary for parametric methods. The association between bioactive lipids and OA was performed using logistic regressions using OA status as the outcome and adjusting for age, sex, BMI, and NSAID use on a sample of 48 knee OA participants versus 44 nonOA controls as listed in Table 1. Adjustment for multiple testing was carried out using a Bonferroni correction. For individuals with SF extracted from more than one knee, the average of both knees was used if both were affected or both were unaffected. The differences in 10 pairs of matched knees with unilateral knee OA was carried out using a paired *t*-test. Association with radiographic progression was carried out by logistic regression adjusted for age, sex, BMI, follow-up time, and OA case-control status at baseline. The progression outcome was defined as a change of one or more in tibiofemoral K/L grade and was analyzed adjusting for age, BMI, sex, time to follow-up, and status at baseline (OA or not). Data from 102 knees were included in this analysis. Statistical analyses were performed using R version 3.0.1 (www.cran.org)

### Genetic association analysis

The TwinsUK registry contains twin volunteers recruited through national media campaigns and from other twin registers (19). The study was approved by the St Thomas’ Hospital research ethics committee, and all participants provided written informed consent. Stored serum samples from 250 samples from individuals from this cohort with genome-wide genotyping were transferred to the School of Pharmacy in Nottingham. Four omega-6 oxylipins (5,6 DHET, 8,9 DHET, 11,12-DHET, and 14,15-DHET) were measured as for the OA samples and levels were correlated to genetic variants in the *EPHX2* gene. To account for family structure in the TwinsUK cohort, we utilized the GenABEL software package (http://www.genabel.org/) (20), which is designed for genetic association analysis of family-based data by incorporating a pairwise kinship matrix calculated using genotyping data in the polygenic model to correct relatedness and hidden population stratification. The linear regression implemented in the software was used to test the association between a given SNP and the four oxylipins.

#### Genetic variation in the EPHX2 gene.

In order to increase statistical power (by reducing the number of tests), we used 20 SNPs that tag 97% of genetic variation with MAF ≥ 5% in this gene with r^2^ > 0.70. The genetic association analysis for the *EPXH2* gene was performed using inverse normal transformations for each of the four oxylipins under investigation. As a result of the transformation, each of the four oxylipins tested had a normal distribution (mean of 0 and standard deviation of 1) across TwinsUK.

## RESULTS

The descriptive characteristics of study participants, radiographic grade, and concentration of the 22 omega-6 lipids investigated are shown in **Table 1**. NSAID users were more likely to have OA in our total case-control sample (73% in cases, 56% in controls) although not significantly so (*P* < 0.13) and had a significantly higher BMI than nonNSAID users [32.1 (SD = 8.4) kg/m^2^ vs. 28.7 (SD = 7.0) kg/m^2^ vs. *P* < 0.049]. Adjusting for age, sex, and BMI, two of the SF oxylipins,16-HETE and 20-HETE, had significantly lower concentrations in NSAID users than nonNSAID users; therefore, all analyses were adjusted for use of NSAIDs.

**TABLE 1.** Descriptive characteristics of study participants and of the knees from which synovial fluid was extracted, including radiographic grade and follow-up time

	Control		Knee OA			
Number of individuals	44				58	
F%	45.9%				60.3%	
age (SD)	67.94	7.58			69.28	8.65
BMI (SD)	28.34	5.38			29.90	6.74
NSAID use	16.3%				36.1%	
Antioxidant vitamins use	14.2%				18.1%	
Plasma total n-6 (mmol/l)	2.55	0.31			2.57	0.45
plasma total n-3 (mmol/l)	0.34	0.05			0.33	0.06
plasma total PUFA (mmol/l)	2.89	0.34			2.90	0.50
plasma AA (mmol/l)	0.30	0.12			0.31	0.13
plasma LNA (mmol/l)	1.59	0.63			1.64	0.85
	Unaffected knees		Unaffected knees		Affected knees	
Number of knees	48		10		67	
K/L at baseline (0/1/2/3/4)	0.95/0.05/0/0/0		0.5/0.5/0/0/0		0/0/0.34/0.38/0.28	
K/L at follow-up (0/1/2/3/4)	0.78/0.12/0.07/0.02/0		0.5/0.3/0.2/0/0		0/0/0.18/0.46/0.36	
Signs of clinical effusion	12.5%		40%		57%	
	mean	SD	mean	SD	mean	SD
Follow up time (years)	2.77	0.78	3.77	1.4	3.52	1.3
5-HETE (pmol/ml)	0.427	0.636	0.258	0.110	0.345	0.295
8-HETE (pmol/ml)	0.465	1.238	0.188	0.112	0.318	0.573
11-HETE (pmol/ml)	0.348	0.602	0.198	0.107	0.241	0.291
12-HETE (pmol/ml)	7.112	28.233	0.222	0.182	5.211	28.790
15-HETE (pmol/ml)	0.106	0.272	0.041	0.026	0.057	0.097
16-HETE (pmol/ml)	0.273	0.201	0.374	0.144	0.365	0.143
19-HETE (pmol/ml)	3.864	15.928	0.970	1.034	2.366	9.119
20-HETE (pmol/ml)	0.819	0.943	0.725	0.604	0.949	0.779
8,9-EET (pmol/ml)	0.561	1.321	0.210	0.154	0.316	0.517
11,12-EET (pmol/ml)	0.292	0.604	0.170	0.082	0.237	0.341
14,15-EET (pmol/ml)	0.650	1.015	0.606	0.317	0.700	0.715
5,6-DHET (pmol/ml)	0.136	0.174	0.084	0.074	0.134	0.207
8,9-DHET (pmol/ml)	0.299	0.200	0.339	0.195	0.368	0.222
11,12-DHET (pmol/ml)	1.195	0.943	1.616	0.757	1.735	0.903
14,15-DHET (pmol/ml)	1.739	1.099	2.032	0.820	2.584	1.087
9-HODE (nmol/ml)	15.523	36.585	2.421	1.337	5.988	17.101
13-HODE (nmol/ml)	17.985	36.461	4.889	2.045	8.120	13.417
9-OxoODE (nmol/ml)	12.962	37.300	2.550	3.249	3.569	6.875
LNA (nmol/ml)	130.764	242.918	41.045	27.699	69.998	76.160
AA (nmol/ml)	19.052	48.678	9.606	6.148	12.125	10.973
LTB4 (pmol/ml)	0.325	1.204	6.482	15.354	0.762	1.795
PGD2 (pmol/ml)	0.016	0.037	0.035	0.049	0.054	0.106

The mean concentration and SDs of the 22 lipids measured for each group is also shown. The distribution of radiographic K/L grade is shown as the proportion with grades 0, 1, 2, 3, and 4 at the tibiofemoral compartment. LTB4, leukotrienes B4.

**Fig. 1.**
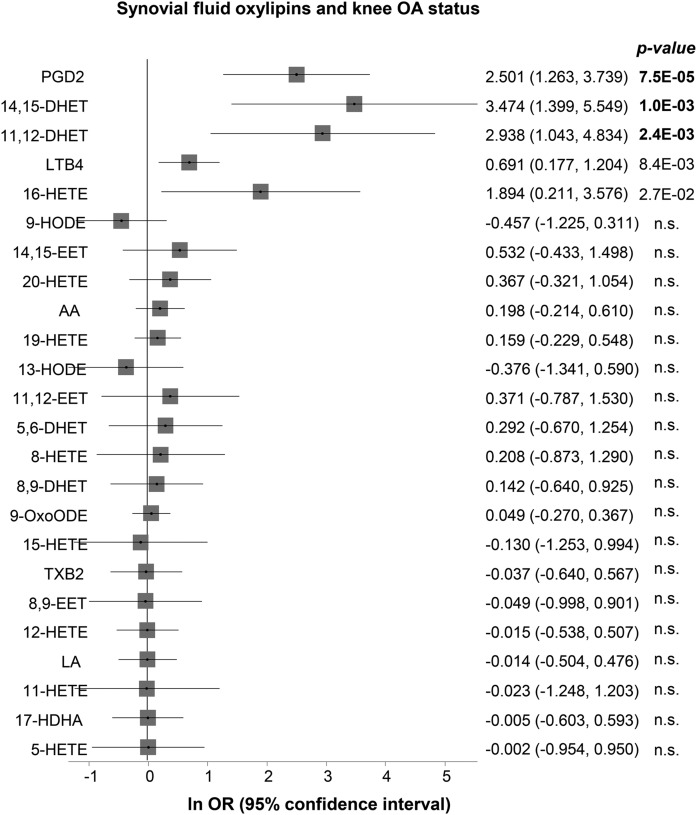
Forest plot showing the logistic regression coefficient (natural logarithm of the odds ratio) adjusted for age, sex, BMI, and use of NSAIDs. The effect is per log10 concentration unit as listed in Table 1.

We first determined the association between plasma levels of total omega-3, total omega-6, AA, and linoleic acid with OA and found no association (Table 1). We then assessed whether there were differences in SF levels of omega-6 lipids and OA status. The coefficients from the logistic regression and the corresponding 95% CI are shown in **Fig. 1**. In our discovery set of 48 OA vs. 44 controls, out of the 22 lipids measured, three oxylipins (prostaglandin D2, 11,12-DHET, and 14,15-DHET) were significantly different between these two groups after adjustment for covariates and multiple testing (Bonferroni threshold of *P* < 0.0023 adjusting for 22 tests). These results remained similar when we further adjusted for use of anti-oxidant vitamins (supplemental Table S1).

**Fig. 2.**
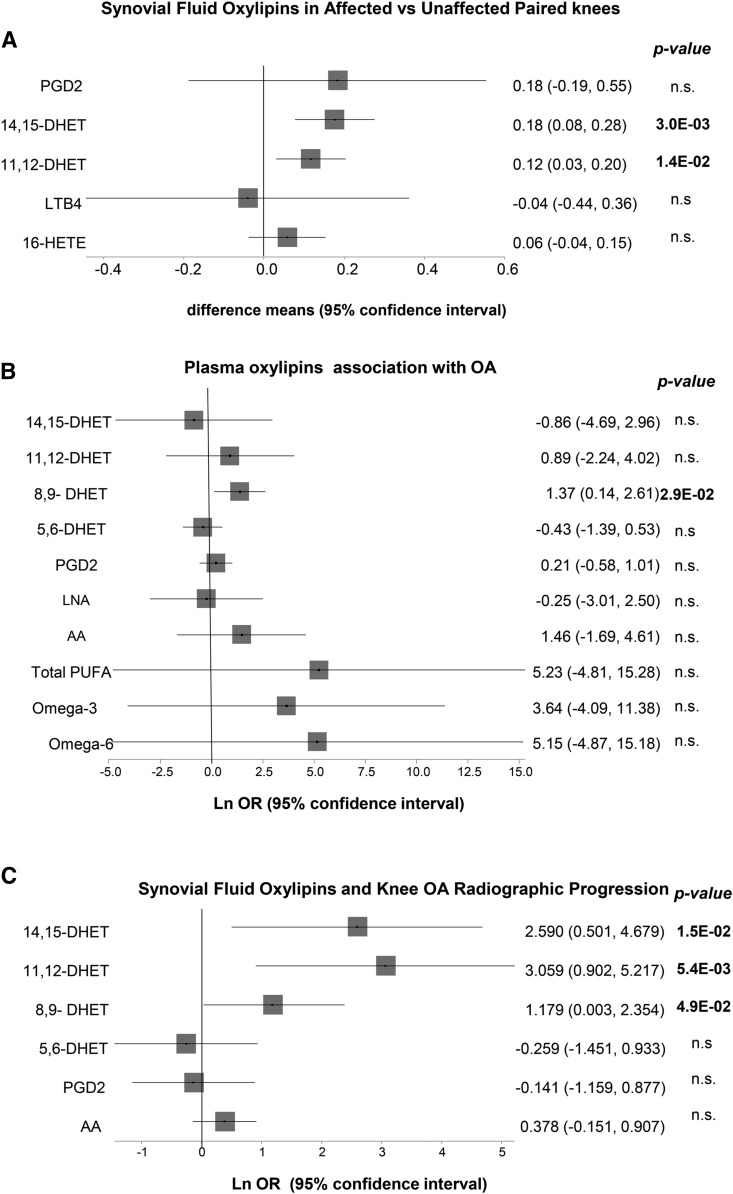
Forest plots showing the association between selected oxylipins. A: Difference between synovial fluid levels in affected and unaffected knees from 10 individuals with knee OA. *P*-values are derived from paired *t*-test analysis. B: Logistic regression coefficients for association between knee OA and plasma levels of total omega-3, total omega-6, AA, PGD2, and the four DHETs, total PUFA oxylipins associated with OA and related compounds. C: Logistic regression coefficients for association between radiographic progression and SF levels of selected oxylipins plus AA. All analyses are adjusted for age, sex, BMI and use of NSAIDs.

Individuals who had SF for one affected and one unaffected knee were analyzed separately and used to validate our observation for the involvement of these three oxylipins in OA. The affected knees had significantly higher concentrations of both 11,12-DHET and 14,15-DHET compared with the unaffected knees of the same individuals (**Fig. 2A**). However, there was no difference in the levels of PGD2 between the affected and unaffected knees; therefore, we did not investigate this compound any further. The same was seen regarding the association between 16-HETE with OA; that is, we saw no difference between affected and unaffected knees from knee OA patients

**Fig. 3.**
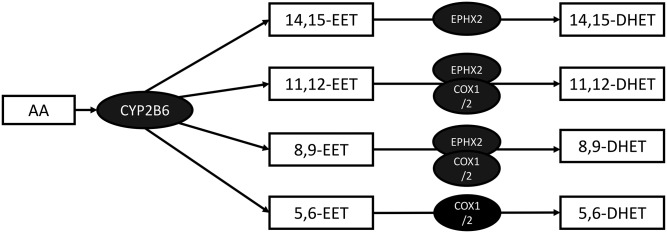
Biochemical pathway leading from AA into DHETs.

Both of the replicated lipids are DHETs. DHETs are metabolites of EETs, which in turn are synthesized from AA by cytochrome P-450 epoxygenases. (**Fig. 3**). The SF levels of two other compounds in the same class, 5,6-DHET and 8,9-DHET, also tested, were not associated with OA (Fig. 1).

We then tested whether circulating levels of the four DHETs were associated with OA, or whether circulating levels of total omega-6, omega-3, or AA were associated with OA. We found that, with the exception of 8,9-DHET, none of the lipid levels are associated with OA status after adjustment for age, sex, BMI, and use of NSAIDs (Fig. 2B). Results remained the same after adjustment for use of antioxidant vitamins (supplemental Table S2).

Given the availability of radiographs taken 3.25 years later for most of these individuals, we investigated whether SF levels of these lipids were also correlated with radiographic progression. Higher concentrations of all three, 8,9-DHET, 11,12-DHET, and 14,15-DHET, were nominally associated with increased risk of tibiofemoral OA progression (Fig. 2C). However, levels of AA, PGD2, and 5,6-DHET were not associated with radiographic progression.

Because the inflammatory state of the knee might be a confounding factor in this analysis, we took into consideration the presence of knee effusion in a secondary analysis. The results are included in supplemental Table S3 and the values are similar to those obtained without adjustment.

DHETs are epoxides of EETs normally generated by soluble epoxide hydrolase (sEH) (21) but can also be epoxidized by COX-1 and COX-2 (Fig. 3). It has been reported in the literature that sEH has the highest affinity for 14,15-EET (22), whereas when EETs are epoxidized by COX-1 and COX-2, the affinities are 8,9-EET > 5,6-EET > 11,12-EET, and 14,15-EET is inactive (23). These facts suggest that the three DHET compounds identified by us to be associated with OA are products of the sEH enzyme, but that the DHET not associated is not derived from sEH. To test this hypothesis, we used genetic data from a separate cohort of healthy individuals, for whom genome-wide genotyping data was available. Polymorphisms in the *EPHX2* gene, which encodes the sEH enzyme, have been previously implicated in the activity of sEH (24, 25), measured by the accumulation of 14,15-DHET.

**Fig. 4.**
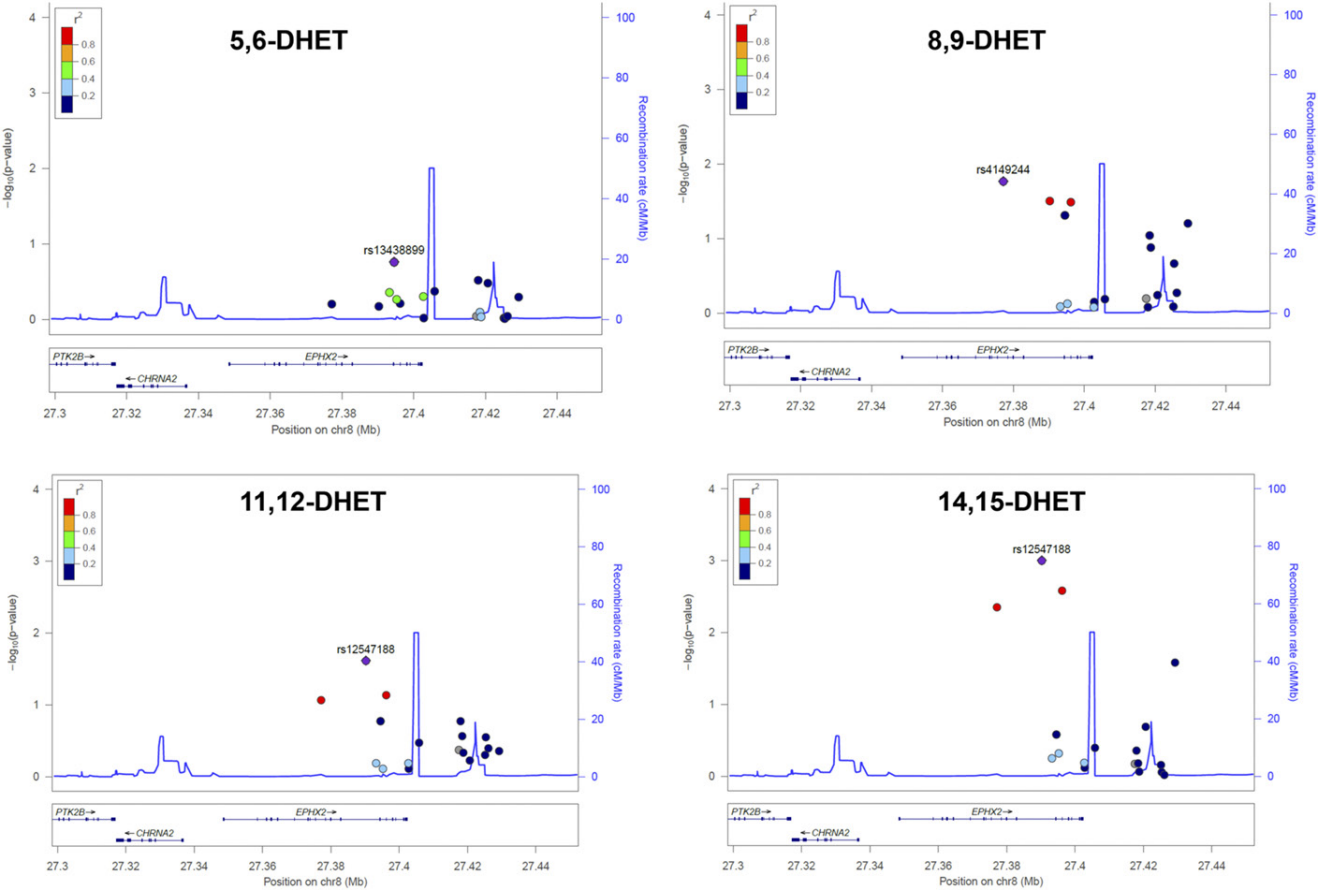
Locus plots showing the association between SNPs at the *EPXH2* gene (encoding sEH) and 5,6-DHET, 8,9-DHET, 11,12-DHET, and 14,15-DHET.

Therefore, we tested for association between serum levels of the four DHET compounds and polymorphisms in the *EPHX2* gene. We found that, whereas variants in the EPHX2 gene are indeed significantly associated with 8,9-DHET, 11,12-DHET, and 14,15-DHET (**Fig. 4B**–D), there is no evidence for association with levels of 5,6-DHET (Fig. 4A), suggesting that the sEH enzyme plays a larger role in the generation of the DHET compounds identified to be associated with OA and OA progression than in the one not associated.

## DISCUSSION

In this study, we investigated in depth the relationship between omega-6 lipids and knee OA and found that compounds generated by sEH are associated both with prevalent knee OA and structural OA progression. On the other hand, we did not find a consistent increase of circulating total omega-6 lipids in OA-affected individuals compared with controls, suggesting that omega-6 compound associations with radiographic damage are not simply reflecting systemic inflammation among individuals with OA. We report three lipids whose concentrations are significantly increased in OA compared with controls in SF. Only two of these were replicated when we looked at affected vs. unaffected knees of 10 individuals; specifically 11,12-DHET and 14,15-DHET. Levels of 11,12-DHET and 14,15-DHET, along with levels of 8,9-DHET, were also increased in knees showing radiographic OA progression compared with knees without radiographic progression, after adjusting for use of NSAIDs. In addition, plasma levels of 8,9 DHET were nominally associated with OA.

The three compounds identified are known to be metabolized from EETs by the sEH enzyme (21), which is a known anti-inflammatory target. Our genetic data suggest that it is the products of sEH in SF that are significantly associated with knee OA prevalence and progression, whereas DHET, which is known to be predominantly generated by COXs, is not associated with OA.

Although originally DHETs were considered to be inactive degradation products, there are reports that some of them have biological activity (26). For example, 14,15-DHET levels correlate with high sensitivity C-reactive protein levels and are significantly higher in people with coronary heart disease (27). Another possibility is that SF levels of 8,9-DHET, 11,12-DHET, and 14,15-DHET may be reflective of activity of the pro-inflammatory sEH enzyme in the joint. If this is confirmed by future studies, this would have important therapeutic implications. Several small molecules have already been developed (21) against this enzyme and it may, therefore, be a pharmacological target to reduce OA onset and progression.

Previous studies on knee OA have suggested that the presence of synovitis seen by arthroscopy, MRI, and ultrasound may predict an increased risk of disease progression (2, 28, 29). A role for synovial inflammation in OA progression has also previously been shown for hand OA (30, 31). The evidence from such studies points to inflammatory activity in the synovium playing a role in cartilage loss in OA joints. It has been hypothesized that the main molecular mediators responsible for this phenomenon might be cytokines and PGs (4), but our data suggest that sEH may be implicated in this inflammatory OA process. In our study, we find that SF levels of 11,12-DHET and 14,15-DHET are significantly associated with progression even after adjustment for knee effusion at baseline. On the other hand, at least in the case of omega-6 lipids, we did not find evidence of lipoxygenase metabolites being implicated in risk of radiographic OA.

We note several study limitations. Although we have been able to replicate the associations between OA, 11,12-DHET, and 14,15-DHET using a subset of unilateral OA individuals, we have not validated our findings in independent populations. Our OA population is derived from secondary care, so generalizability of findings may be limited. Also, we used plain radiographs and focused on the tibiofemoral compartments, whereas other imaging methods such as MRI would have been more sensitive to change in OA features. Nonetheless, our study explores for the first time the specific omega-6 oxylipins that are involved in OA and OA progression and generates a useful hypothesis to test regarding the role of sEH in OA pathogenesis. Further research is required to find out whether blocking the expression of the sEH gene associates with a reduction in OA progression.

## Supplementary Material

Supplemental Data
