# Supplementary material for: Omega-6 oxylipins generated by soluble epoxide hydrolase are associated with knee osteoarthritis
Source: J Lipid Res. 2018 Jul 9;59(9):1763–70. doi: 10.1194/jlr.P085118 (PMC6121933; doi:10.1194/jlr.P085118)
Supplement: Supplemental Data [file supp_59_9_1763__index.html]

Omega-6 oxylipins generated by soluble epoxide hydrolase are associated with knee osteoarthritis — Supplemental Data 

# Omega-6 oxylipins generated by soluble epoxide hydrolase are associated with knee osteoarthritis

## Supplemental Data

- Supplemental Table S3 (.docx, 14 KB) - Association between radiographic progression SF data OA adjusted for age, sex, BMI, use of time to follow-up, presence of effusion at baseline and use of antioxidant vitamins.
- Supplemental Table S2 (.docx, 15 KB) - Association between plasma levels of various polyunsaturated fatty acids and knee OA adjusted for age, sex, BMI, use of NSAIDs and use of anti-oxidant vitamins
