# Supplementary material for: Omega-6 oxylipins generated by soluble epoxide hydrolase are associated with knee osteoarthritis
Source: J Lipid Res. 2018 Jul 9;59(9):1763–70. doi: 10.1194/jlr.P085118 (PMC6121933; doi:10.1194/jlr.P085118)
Supplement: Supplemental Data [file 10.1194_P085118_jlr.P085118-1.docx]

**Supplemental Table S3** Association between radiographic progression SF data OA adjusted for age, sex, BMI, use of time to follow-up, presence of effusion at baseline and use of antioxidant vitamins.

| **Omega-6 lipid** | **ln OR** | **95%CI** | **p-value** |
| --- | --- | --- | --- |
| 14,15-DHET | 0.447 | [0.095 - 0.799] | **0.013** |
| 11,12-DHET | 0.443 | [0.100 - 0.785] | **0.011** |
| 8,9-DHET | 0.176 | [-0.020 - 0.373] | 0.079 |
| 5,6-DHET | 0.447 | [-0.261 - 0.229] | 0.896 |
| AA | 0.030 | [-0.069 - 0.130] | 0.550 |
| PGD2 | 0.140 | [-0.063 - 0.344] | 0.177 |
