# Supplementary material for: Omega-6 oxylipins generated by soluble epoxide hydrolase are associated with knee osteoarthritis
Source: J Lipid Res. 2018 Jul 9;59(9):1763–70. doi: 10.1194/jlr.P085118 (PMC6121933; doi:10.1194/jlr.P085118)
Supplement: Supplemental Data [file 10.1194_P085118_jlr.P085118-2.docx]

**Supplemental Table S2**. Association between plasma levels of various polyunsaturated fatty acids and knee OA adjusted for age, sex, BMI, use of NSAIDs and use of anti-oxidant vitamins

| **Plasma lipid** | **lnOR** | **95% CI** | **p-value** |
| --- | --- | --- | --- |
| 14,15-DHET | 0.106 | [-0.506 - 0.719] | 0.73 |
| 11,12-DHET | 0.115 | [-0.378 - 0.607] | 0.65 |
| 8,9-DHET | 0.275 | [0.082 - 0.468] | **0.0052** |
| 5,6-DHET | 0.020 | [-0.126 - 0.166] | 0.79 |
| PGD2 | 0.035 | [-0.111 - 0.181] | 0.64 |
| LNA | 0.022 | [-0.520 - 0.564] | 0.94 |
| AA | 0.000 | [-0.487 - 0.487] | 1.00 |
| total PUFA | 0.479 | [-1.235 - 2.193] | 0.58 |
| Omega-6 | 0.504 | [-1.200 - 2.209] | 0.56 |
| Omega-3 | 0.220 | [-1.130 - 1.570] | 0.75 |
